# The Impact of Formulation on Lutein, Zeaxanthin, and *meso*-Zeaxanthin Bioavailability: A Randomised Double-Blind Placebo-Controlled Study

**DOI:** 10.3390/antiox9080767

**Published:** 2020-08-18

**Authors:** Marina Green-Gomez, Alfonso Prado-Cabrero, Rachel Moran, Tommy Power, Laura G. Gómez-Mascaraque, Jim Stack, John M. Nolan

**Affiliations:** 1 Nutrition Research Centre Ireland, School of Health Sciences, Carriganore House, Waterford Institute of Technology West Campus, X91 X236 Waterford, Ireland; mgreen@wit.ie (M.G.-G.); aprado-cabrero@wit.ie (A.P.-C.); rachelmoran.mprg@gmail.com (R.M.); tbpower@wit.ie (T.P.); jimstack434@gmail.com (J.S.); 2 Teagasc Food Research Centre, Moorepark, Fermoy, P61 C996 Co. Cork, Ireland; laura.mascaraque@teagasc.ie

**Keywords:** *meso*-zeaxanthin, zeaxanthin, lutein, carotenoid diacetates, bioavailability, micromicelles

## Abstract

Lutein (L), zeaxanthin (Z), and *meso*-zeaxanthin (MZ) have been the focus of research and commercial interest for their applications in human health. Research into formulations to enhance their bioavailability is merited. This 6 month randomised placebo-controlled trial involving 81 healthy volunteers compared the bioavailability of different formulations of free L, Z, and MZ in sunflower or omega-3 oil versus L, Z, and MZ diacetates (Ld, Zd, and MZd) in a micromicellar formulation. Fasting serum carotenoids, macular pigment, and skin carotenoid score were analysed at baseline and 6 months. Serum L, Z, and MZ concentrations increased in all active interventions compared to placebo (*p* < 0.001 to *p* = 0.008). The diacetate micromicelle formulation exhibited a significantly higher mean response in serum concentrations of Z and MZ compared to the other active interventions (*p* = 0.002 to 0.019). A micromicellar formulation with solubilised Z and MZ diacetates is a promising technology advancement that enhances the bioavailability of these carotenoids when compared to traditional carotenoid formulations (ISRCTN clinical trial registration number: ISRCTN18206561).

## 1. Introduction

Lutein (L), zeaxanthin (Z), and *meso*-zeaxanthin (MZ) are xanthophyll carotenoids (XC) that singularly deposit in the human macula lutea [1], where they are known as macular pigment (MP). L and Z are obtained solely through dietary intake [2]. MZ has been proposed to be obtained from the endogenous conversion of L in the retinal pigment epithelium [3], but it can be also found in trace amounts in diet [4]. Over the last two decades, intervention trials have studied the role of L, Z, and MZ in human health using nutritional supplements [2]. Reports confirmed that these carotenoids enhance visual performance [5,6,7,8,9,10,11,12] and cognitive function [13], and are potential preventive and therapeutic agents in retinal pathology, such as non-advanced age-related macular degeneration (AMD) [14].

**Figure 1:**
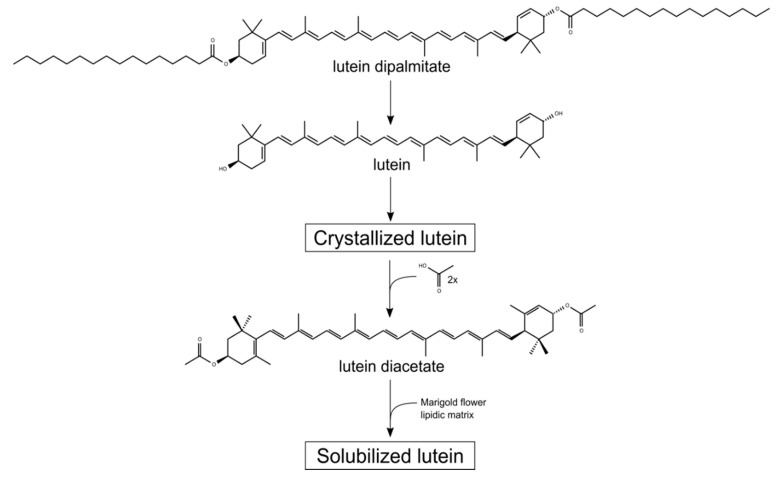
Formation of lutein (L) from marigold flower as free L (microcrystals) and as a diacetate derivative. In the flower, L is present as esterified L with fatty acids. To extract this carotenoid, it is de-esterified and purified by crystallisation. To solubilise these crystals and facilitate absorption in the digestive system, L and other xanthophyll carotenoids (XCs) can be re-esterified with acetate or propionate upon crystallisation and re-suspended in the flower’s lipid matrix with added surfactants to maintain the XCs solubilised under ambient conditions.

L used in nutritional supplements is extracted from the marigold flower (*Tagetes erecta* L.) [15], while Z is obtained from specific varieties of this flower [16] and peppers [17]. MZ is obtained from L through a process that promotes the migration of a double bond that turns the ε-ring of L into a β-ring [18]. In every case, the final purification step forms XC microcrystals [17,19] (Figure 1). Nutraceutical companies continually seek to develop new methods to protect these microcrystals from oxidation, improve their solubility in aqueous matrices, and increase their bioavailability in the digestive system. Among the methods described to protect the XC microcrystals are the dispersion in edible oils, encapsulation with biopolymers [20] or mechanochemical complexation [21,22]. To increase bioavailability and solubility in different matrices, researchers have emulsified the XC following different methods [23,24]. However, none of these methods manage to dissolve the microcrystals completely. Recently, a new method esterifying the XCs with short organic acids, claimed to keep XCs solubilised without the formation of microcrystals under environmental conditions of temperature and pressure [23,25]. In this process, XCs are esterified with acetate or propionate to form L, Z, and MZ diacetates (Ld, Zd, and MZd, respectively). After this reaction takes place, XC derivatives are then homogenised in their natural original flower matrix in the presence of lipids, phospholipids, fatty acids, and emulsifiers to keep XCs soluble. In the digestive system, this soluble state facilitates the incorporation of XCs into micromicelles, which are spherical aggregates of lipid molecules in the presence of amphiphilic compounds known as surfactants [24,26]. This formulation has been previously tested [27,28,29] in clinical trials and compared to crystallised formulations (free lutein).

Here, we present the findings of the Carotenoid-Omega Availability Study (COAST), which was performed to compare the bioavailability of Ld, Zd and MZd in a micromicelle formulation with classical formulations containing the free carotenoids as microcrystals suspended in oil.

## 2. Materials and Methods

### 2.1. Design and Study Population

COAST was a 6 month, double-blind, block-randomised placebo-controlled study involving 81 healthy participants between 18 and 65 years old. Participant recruitment and assessment commenced in December 2017 and ended in December 2018. Recruitment was achieved through local media, and advertisement at the Waterford Institute of Technology, local fitness centres, and with employees of an industry based in Waterford, Ireland. Participants were excluded if they had a medical diagnosis of a critical or acute medical condition and/or if they were taking nutritional supplements containing L, Z, MZ, or omega-3 fatty acids. Every participant enrolled in the study provided written informed consent prior to commencement. The study protocol was approved in May 2017 by the Research Ethics Committees of the Waterford Institute of Technology (Waterford, Ireland; +353-51-302000; ethics@wit.ie), ref: 17/HS-MPRG/01 and the HSE, South Eastern Area (University Hospital Waterford, Waterford, Ireland; +353-51-842026). Industrial Organica, S.A. de C.V., the manufacturer of the nutritional supplements, had no role in the design of the study, the collection and analysis of the data, or the preparation of the manuscript. All authors vouch for the accuracy of the data and the fidelity of the study to the protocol.

### 2.2. Interventions

COAST was a five-arm intervention study, where participants were randomly allocated, with equal probability and separately for men and women, to one of four active intervention groups or to a placebo group. Label claims of the nutritional content in the intervention supplements were as follows: Group 1, L (10 mg) + MZ (10 mg) + Z (2 mg) provided in one capsule; Group 2, L (10 mg) + MZ (10 mg) + Z (2 mg) provided in two capsules; Group 3, L (10 mg) + MZ (10 mg) + Z (2 mg) provided in DHA (430 mg) and EPA (90 mg) in two capsules; and Group 4, Ld (10 mg) + MZd (10 mg) + Zd (2 mg) provided in a micromicelle formulation in one capsule; or Group 5, placebo (sunflower oil). L, Z, and MZ were supplied in free form as microcrystals for Groups 1,2, and 3. For Group 4, L, Z and MZ were supplied as L, Z, and MZ diacetates. In Group 1 and 2, carotenoids were provided in a sunflower oil suspension; in Group 3, carotenoids were suspended in fish oil supplied by Epax (Ålesund, Norway; product number: EPAX1050TG), and vitamin E (DL-α-tocopheryl acetate; 5 g/kg) was added as a preservative; and in Group 4, carotenoids were provided as a solubilisate prepared for micellarisation (marketed as MicroMic™). The supplements were provided to the participants in a sealed container and the capsules for all the intervention groups were identical in appearance. Subjects were instructed to take either 1 or 2 capsules per day depending on the intervention with a meal. The supplements were provided by Industrial Organica (Monterrey, Mexico) free-of-charge for use in the trial.

#### Analysis of Supplements

Carotenoid analysis of the supplements used in this study was conducted at our laboratory as previously described [30]. For carotenoid content of the formulation containing diacetate-carotenoids, the mobile phase was adjusted to hexane:isopropanol 99:1 (*v*/*v*). The carotenoid profile of the oil phase of supplements was conducted after filtering the content of five capsules through two nylon filters of 0.45 μm pore diameter (Chromafil, Apex Scientific Ltd., Kildare, Ireland). Capsule contents were also analysed under optical and polarised light microscopy. Images were taken using an Olympus BX51 digital microscopy system (Olympus Corporation, Tokyo, Japan) equipped with a polariser and a ProgRes CT3 digital camera head (Jenoptik, Jena, Germany). Micrographs were obtained using a 60× oil immersion objective. ProgRes CapturePro software (v 2.10.0.0) was used for image capturing.

### 2.3. Study Evaluations

#### 2.3.1. Demographic, Lifestyle, Medical, and Dietary Assessment

Standardised case report forms were used to record demographics, lifestyle, medical history and anthropometrics at two timepoints, at baseline and at 6 months following supplementation. Cigarette smoking was recorded by smoking status as follows: never, if never smoked more than 100 cigarettes, former, if smoked more than 100 cigarettes in the past year and none in the last month; or current. Education was recorded as high school or less, bachelor’s degree, or postgraduate education. Physical examination included height and body weight to calculate body mass index (BMI, kg/m^2^). International cut-offs for normal, overweight, and obesity were used.

#### 2.3.2. Outcome Measures

L, Z, and MZ bioavailability measured as a response in serum and tissue concentrations was the primary outcome of the present study. Serum carotenoid concentrations were analysed as the total concentrations (μmol/L) for each of carotenoids in serum. Total carotenoid concentrations were obtained by the sum of L, Z, and MZ concentrations. Tissue concentrations of L, Z, and MZ were measured as a composite MP and skin carotenoid score. All methods are described below. Outcome variables were recorded at baseline and at 6 months.

#### 2.3.3. Macular Pigment Measurement

MP was measured by dual-wavelength autofluorescence (AF) using the Spectralis investigational macular pigment optical density (MPOD) module (Heidelberg Engineering GmbH, Heidelberg, Germany). Specifications and details on the technique and image acquisition have been described elsewhere [30]. In short, pupils were dilated prior to MP measurement, and patient details were entered into the Heidelberg Eye Explorer (HEYEX version 1.7.1.0) software. Alignment, focus and camera sensitivity were first optimised in near-infrared reflectance mode. Subsequently, BAF+GAF (simultaneous blue and green AF) movie images were acquired, while the HEYEX software ensured proper alignment and averaging of these images in order to generate a MP density map, where the reference eccentricity was defined at 7° retinal eccentricity from point of fixation (where MPOD was defined as zero). MP measurement was reported in terms of MPOV as standardised previously [30].

#### 2.3.4. Skin Carotenoid Concentrations

Carotenoid concentrations in the skin were obtained using the Nu Skin Pharmanex S3 scanner, a non-invasive instrument that uses Raman spectroscopy technology [2]. This technique generates a skin carotenoid score (SCS) by measuring skin carotenoid concentrations between the maximal and distal palmar creases, directly below the fifth finger of the right hand using the Pharmanex BioPhotonic Scanner device.

#### 2.3.5. Carotenoid Serum Concentrations

Fasting (overnight fast > 9 h) blood samples were collected at 0, 3, and 6 months for XC serum analysis. Blood samples were collected by standard venepuncture techniques in 9 mL blood collection tubes (BD Vacutainer SST Serum Separation Tubes) containing a “Z Serum Sep Clot Activator”. Collection tubes underwent thorough mixing of the clot activator. The blood samples were left for 30 min at room temperature to clot and then centrifuged at 725 g for 10 min in a GruppeGC12 centrifuge (Desaga Sarstedt) to separate the serum from the whole blood. Following centrifugation, serum was transferred to light-resistant microtubes and stored at circa −80 °C until the time of batch analysis. Serum carotenoid analysis was performed by high performance liquid chromatography (HPLC), using a method previously described by our laboratory [31]. The calibration lines used, as well as the lower and upper limits of quantification (LLOQ and ULOQ respectively), were as in the cited work. Serum carotenoid analysis was completed in sixteen independent batches, with a maximum intra-day precision of 7.28%, measured as RSD, and an inter-day precision of 3.16% (RSD).

### 2.4. Follow-Up and Adherence

Follow-up study visits were scheduled at 3 months after baseline and at 6 months (endpoint). Adherence to the treatment regime was assessed by pill count at each visit and by serum analysis at 3 months. Information on their change in lifestyle and health as well as adverse events was collected at each visit. Adverse events were collected through a non-validated questionnaire.

### 2.5. Statistical Analysis

Data were described using usual statistics, including means (±SDs), medians, minimum, and maximum values for quantitative variables, and frequencies and percentages for qualitative variables. Between-group differences at baseline were analysed using analysis of variance, or Kruskal–Wallis, as appropriate for quantitative variables, and chi-square test for qualitative variables. Groups differed significantly with respect to BMI at baseline, which was further controlled using ANCOVA. However, BMI in these models was not significantly related to any outcome variable, so it was subsequently removed from each model. The results reported below, therefore, are all for simpler models, relating change in outcome variables to intervention alone.

General linear models were used to analyse the change in primary outcome variables (change carotenoid serum concentrations, MPOV and skin carotenoid score). Change was analysed as the difference of the outcome variable from baseline and 6 months. The hypotheses were (a) that the active intervention groups (unrelated treatments) would all have a higher average response after six months, in serum and in tissue concentrations compared with the placebo group, and (b) that the diacetate micromicellar formulation would have a higher average response as compared with the other active interventions. The first of these hypotheses was investigated directly from the fitted linear models, and the second using pairwise comparisons based on 2-tailed independent samples T-tests. No adjustment for multiple comparisons was deemed appropriate. Pearson’s coefficient was used to investigate relationships between change in serum and change in tissue of carotenoid concentrations. The statistical package IBM SPSS version 25 (Armonk, NY, USA) was used, and a 5% significance level was applied throughout.

## 3. Results

**Figure 2:**
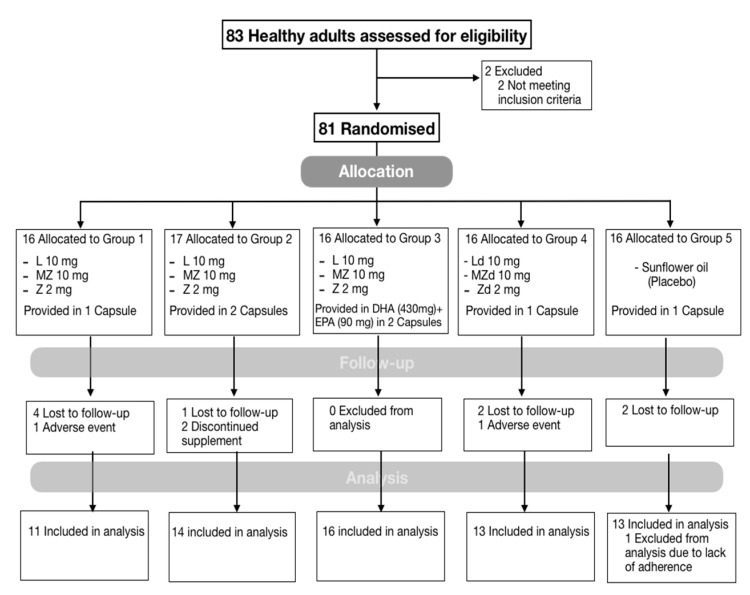
Screening, randomisation, and follow-up of study participants. Abbreviations: L, lutein; MZ, *meso*-zeaxanthin; Z, zeaxanthin. A total of 2 participants discontinued the interventions due to adverse events related to gastrointestinal symptoms, bloating and gastric discomfort when taken in a fasted state. Lost to follow-up was due to loss of contact.

A total of 81 participants were enrolled at baseline with 68 (84%) participants completing final assessment at 6 months; nine (11%) participants were lost to follow-up and four (5%) discontinued the study, one due to pregnancy, two due to minor adverse events, and one due to a general practitioner request (Figure 2). Adverse events reported throughout the 6 months of the study were all related to minor gastrointestinal symptoms: bloating, acid reflux and discomfort. There was no statistical difference between active interventions and placebo (*p* > 0.05). One participant allocated to the placebo arm was excluded from analysis as he reported supplementation with carotenoids during the duration of the study, which was confirmed by the detection of high concentrations of MZ in serum at 6 months.

All the participants who completed the study consumed in average 89% of the assigned number of tablets. There were no significant differences in tablet count between groups (*p* = 0.512). Participants tested at 3 months had an average increase in their serum concentrations of L and Z by 239% and 37%, respectively, and MZ serum concentrations increased from 0 to 0.075 μmol/L.

### 3.1. Baseline Data

The mean (range) age of the participants was 44.8 (25 to 62) years, and 47.8% (n = 32) were women. Baseline characteristics were statistically comparable across the five groups, except for BMI (*p* = 0.027), which was within the normal range in Group 2 and Group 5, but higher for Group 1, Group 3, and Group 4 (Table 1).

The baseline serum and tissue levels of study nutrients were balanced across the treatment groups (Table 1). MZ concentrations in all participants were undetectable at baseline, which confirms the exclusion criterion of MZ supplementation.

### 3.2. Analysis of Carotenoids in Supplements

The biochemical analysis of the supplements used in this study showed different carotenoid concentrations to those in the label claim (Table 2). The statistical analysis conducted to compare results of the analysed concentrations to those in the label claim did not show significantly different results. Therefore, we decided to present the dosages of the formulations as stated by the label claim.

**Figure 3:**
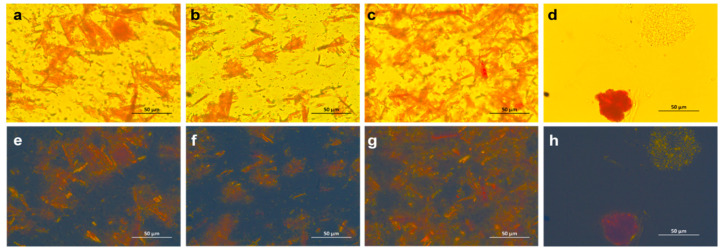
Micrographs of formulations. (**a**–**d**) formulations used in Groups 1, 2, 3 and 4, respectively, analysed by light microscopy, and (**e**–**h**) formulations used in Groups 1, 2, 3 and 4, respectively, analysed by polarised light microscopy.

Light microscopy analysis showed the presence of needle-shaped microcrystals in Groups 1, 2, and 3 but the absence of them in Group 4 (Figure 3a–d). This was further confirmed under polarised light microscopy (Figure 3e–h). To assess the carotenoid profile of the oil phase of the capsules containing microcrystals, we separated the oil phase from the microcrystals in capsules of Group 2. The analysis of the complete content of the capsule (including the microcrystals) showed the presence of the three carotenoids (Supplementary Materials Figure S1A); however, the oil contained almost exclusively L (Supplemental Figure S1B). This content of L accounted for roughly 1.1% of the total L in the capsule.

### 3.3. L, Z, and MZ Serum Concentrations

The increase in serum concentrations of L, Z, and MZ in all active groups was statistically significant compared to placebo (*p* < 0.001 to *p* = 0.008) (Table 3), except for Group 2 (*p* = 0.366).

**Figure 4:**
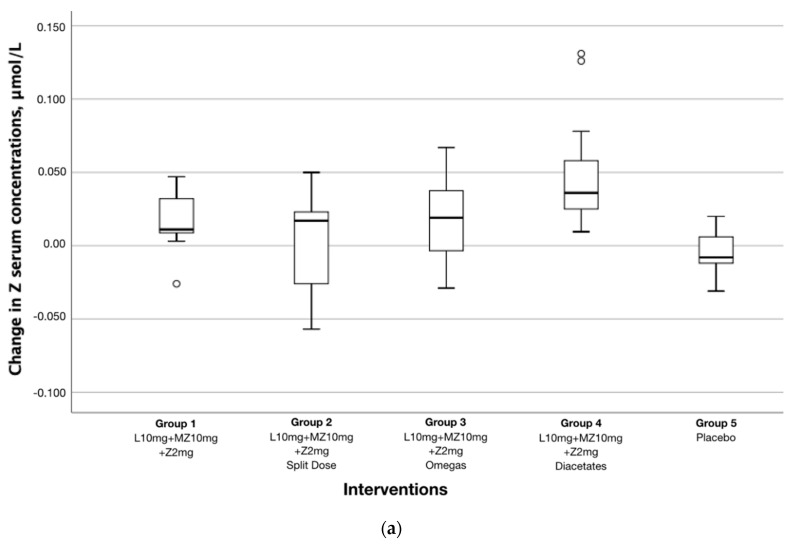

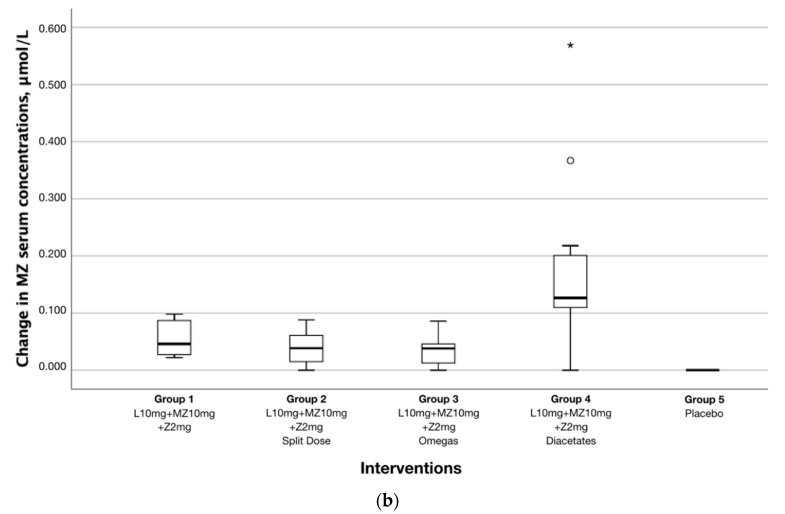
Effect of different formulations in change of serum concentrations. Between-group differences in change in (**a**) Z and (**b**) MZ serum concentrations expressed as change from baseline and 6 months. Z and MZ serum response in Group 4 was significantly higher compared to the other active interventions and placebo (*p* < 0.000 to *p* = 0.019). Outliers are marked with a circle (O) and extreme outliers are marked with an asterisk (*) on the boxplot

In addition, the increase in Z and MZ serum concentrations in Group 4 (diacetate micromicelle formulation) was significantly greater compared to the other three active groups (*p* = 0.002 to 0.019) (Table 4, Figure 4). Table 4 compares the effect of Group 4 on serum and tissue response compared to the other interventions.

### 3.4. L, Z, and MZ Tissue Concentrations

#### 3.4.1. MP and MPOV

**Figure 5:**
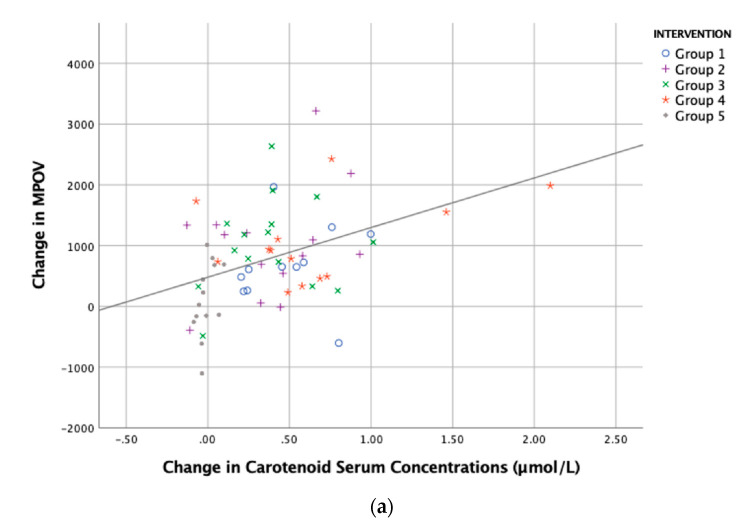

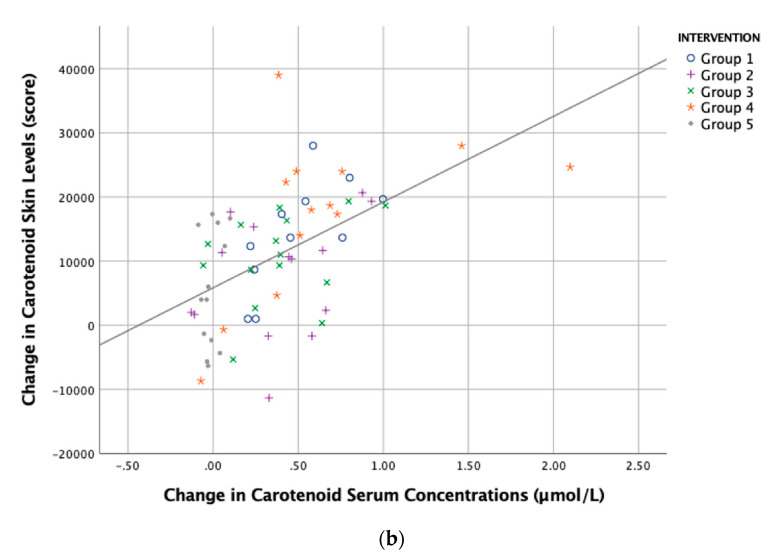
The relationship between the change in carotenoid serum concentration and change in tissue (response). Linear regression analyses of total carotenoid serum concentrations (L + Z + MZ) and (**a**) MPOV (r = 0.408, *p* = 0.001), and (**b**) carotenoid skin score (r = 0.528, *p* < 0.001). Interventions are as follows: Group 1, L (10 mg) + MZ (10 mg) + Z (2 mg) provided in one capsule; Group 2, L (10 mg) + MZ (10 mg) + Z (2 mg) provided in two capsules; Group 3, L (10 mg) + MZ (10 mg) + Z (2 mg) provided in DHA (430 mg) and EPA (90 mg) in two capsules; and Group 4, Ld (10 mg) + MZd (10 mg) + Zd (2 mg) provided in one capsule; or Group 5, placebo (sunflower oil).

The increase in MPOV in all groups was statistically significant compared to placebo (Table 3). Interestingly, the correlation between the change in total serum carotenoids and MPOV was r = 0.408, *p* = 0.001 (Figure 5). There were no significant differences between the four active intervention groups when comparing MPOV improvements.

#### 3.4.2. Carotenoid Skin Concentrations

The change in skin carotenoid concentrations was positively correlated with the total change in serum concentrations (r = 0.528, *p* < 0.001) (Figure 5). The change in skin carotenoid concentrations was statistically significant only in Group 1 and Group 4 compared to placebo (*p* = 0.024 and *p* = 0.012, respectively) (Table 3). In addition, increases in skin carotenoid score in Group 4 were significantly higher compared to Group 2 (*p* = 0.034) (Table 4).

## 4. Discussion

In this multiple-arm, randomised clinical trial, daily supplementation with L, Z, and MZ using different formulations significantly increased serum concentrations of these nutrients compared to placebo. After 6 months of supplementation, the mean serum concentrations of L and Z increased by 193% and 37%, respectively. This dose–response effect is consistent with the ratio of L and Z provided in the supplement (i.e., circa 5:1). In addition, MZ serum concentrations significantly increased from baseline. These percentage increases in serum were comparable to previous studies using similar carotenoid formulations and amounts [12,32]. For example, L increased by 200% in the AREDS 2 study [32], and by 304% in the CREST AMD study [12].

A finding that merits discussion is the impact of the diacetate micromicelle formulation on the absorption of the ingested carotenoids. In the present study, the serum response to Zd and MZd (Group 4, solubilised acetate-esterified XC) was significantly greater compared to the formulations containing free carotenoids. However, it was striking to see that the serum response to Ld remained similar to that of free L. This, however, is consistent with a previous clinical trial performed by Landrum et al., which reported that serum response to Ld was slightly higher but not statistically different when compared to the group supplemented with free L provided as microcrystals in oil [28]. Interestingly, the results of a study in hens conducted by our group to assess the bioavailability of XC diacetates are similar to the present work, as Zd and MZd exhibited a greater capability to increase the deposition of Z and MZ in egg yolk than the free form of these carotenoids [33]. Again, the increase achieved by Ld was similar to the increase observed with free L.

The formulation used in Group 4 contained acetate-esterified XCs plus a series of lipids and surfactants that keep these carotenoid derivatives solubilised in the capsule, without forming microcrystals [23]. These pre-solubilised XCs would be ready for micelle formation in the digestive system for absorption in the intestinal mucosa. On the other hand, free carotenoids form crystals which have to be solubilised by the digestive system prior to the incorporation into micelles [34]. This advantage of pre-solubilised acetate-esterified XCs could explain the greater efficiency of Zd and MZd in increasing serum Z and MZ levels when compared to the microcrystalline form of these carotenoids. It is striking, therefore, that this theoretical superiority of acetate-esterified XCs over microcrystals is appreciated for Zd and MZd, but not for Ld. Multiple mechanisms may be preventing Ld from facilitating an increased absorption. For example, L contains an ε-ring that is oriented differently from the β-ring of Z and MZ, which seems to affect the position that this XC occupies in lipid membranes [35]. Ld, with an acetate group added to the ε-ring, could be positioned less favourably than Zd and MZd in nascent micelles, which could limit its processing by the carboxyl ester lipase (CEL) and subsequent contact with the scavenger receptor class B type 1 (SRB- 1) for internalisation in the intestinal cells. On the other hand, to explain the different behaviour of Ld we also suggest an alternative hypothesis: L microcrystals could be sufficiently solubilised in the digestive tract, thus efficiently yielding soluble free L for micelle formation. In this way, Ld would not offer any advantage over L microcrystals, unlike what we have seen with Zd and MZd. The analysis of one of the formulations containing free carotenoids as microcrystals shows that L has a greater tendency to dissolve in the oil when compared to Z and MZ, which supports this hypothesis. Nevertheless, it would be necessary to understand the physicochemical behaviour of the crystalline form of these xanthophylls in the digestive system to test this hypothesis.

In tissue, after 6 months of supplementation, the mean MPOV significantly increased by 33% on average for all interventions compared to placebo, but improvement over time between the active interventions was not significantly different (given that MPOV improved in all interventions) (Table 3). However, it should be noted that the largest increase in MPOV was seen in Groups 2, 3 and 4, which exhibited almost double the MPOV increases than Group 1. Nevertheless, Group 4, which had the lowest concentrations of carotenoids in the formulation (Table 1), exhibited the largest response (Table 3). A longer duration of supplementation is required to assess the long-term differences between these interventions in terms of MPOV response and functional benefits in vision. With respect to the skin carotenoid score, statistically significant improvements compared to placebo were seen in Groups 1 and 4 only; however, Group 4 was significantly superior to Group 2 (Table 4). This finding is likely attributable to the enhanced bioavailability of Zd and MZd achieved in the micromicelle formulation.

We want to highlight that in the present work, the increase over time in the XC concentrations significantly correlated in serum and tissue for all the groups (r = 0.408, *p* = 0.001 and r = 0.528, *p* < 0.001, respectively). Of note, this is an important result because in our previous interventional trials the change in serum carotenoids poorly correlated with a change in MP, something that is also seen in blood/retinal non-responders [36,37].

The present work describes the behaviour of a diacetate formulation in a solubilisate prepared for micellarisation of L, Z, and MZ compared with crystalline formulations. This study was a double-blind placebo-controlled trial providing a high quality of evidence to the field of nutritional supplements assessed in a multidisciplinary approach. Our findings provide additional evidence for XC bioavailability. The improved response to Zd and MZd is timely given the recent work by Binxing et al., where it appears that Z and MZ are preferentially accumulated in the human retina over L [38]. However, we still acknowledge the importance of the three carotenoids, including L, which collectively contribute to the formation of MP. Future studies should evaluate how these carotenoids become available for absorption in the intestinal tract, their transport in serum, and their absorption in tissue. In addition, a study to separate the effects from both the micellar solubilisate and the acetylation of the aforementioned formulation is needed in order to understand the role of each factor in the bioavailability of these carotenoids.

One limitation of the present study was that in order to compare multiple interventions, the sample size in each group had to be reduced. Even though a multiple-arm RCT design helps to overcome sample limitations, each group had a small sample size, with only one group having >15 participants (Group 3). In addition, other studies reported change over time in serum and tissue over longer periods of time, and these reports suggested that sustained supplementation with carotenoids is required to achieve maximal improvements in MPOV and functional outcomes [39]. However, even though a longer study is likely to have shown a greater difference between groups in MPOV, in this 6 month intervention we did demonstrate significant improvements in MPOV (for all Groups) and skin carotenoid score (for Groups 1 and 4) compared to placebo. The other challenge faced in clinical studies such as this is compliance. Even though we complied with RCT guidelines by counting tablets, we additionally measured XC concentrations in serum at 3 months as an additional compliance marker. Finally, the participants in our study were healthy Irish adults without known established diseases. Thus, we do not know whether these results would be similar in other ethnic groups, diseases, or children, though we speculate that the same biologic mechanisms are operative.

## 5. Conclusions

In conclusion, Z and MZ diacetates in a micromicellar formulation presented an increased bioavailability, most likely due to improved micellarisation and absorption efficiency. This formulation is a promising technology advancement that enhances the bioavailability of Z and MZ when compared to traditional carotenoid formulations.

## Figures and Tables

**Table 1:** Baseline characteristics of the study participants ^1^.

Variable	All Subjects (*n* = 67)	Subjects Divided by Intervention Group				
		Group 1 (*n* = 11)	Group 2 (*n* = 14)	Group 3 (*n* = 16)	Group 4 (*n* = 13)	Group 5 (*n* = 13)
Age (y)	44.8 ± 10	46.0 ± 9.4	44.9 ± 8.9	41.6 ± 10.7	46.3 ± 12.2	46.1 ± 9.7
Females, No. (%)	32 (47.8)	5 (45.5)	7 (50)	8 (50)	5 (38.5)	7 (53.8)
Smoking, No. (%)						
Never	32 (47.8)	5 (45.4)	7 (50.0)	8 (50)	5 (38.5)	7 (53.8)
Former	25 (37.3)	4 (36.4)	4 (28.6)	6 (37.5)	7 (53.8)	4 (30.8)
Current	10 (14.9)	2 (18.2)	3 (21.4)	2 (12.5)	1 (7.7)	2 (15.4)
Education, No. (%)						
High-school	29 (43.3)	2 (18.2)	5 (35.7)	7 (43.7)	10 (76.9)	5 (38.4)
College	25 (37.3)	7 (63.6)	4 (28.6)	6 (37.5)	2 (15.4)	6 (46.2)
Postgraduate	13 (19.4)	2 (18.2)	5 (35.7)	3 (18.8)	1 (7.7)	2 (15.4)
BMI [range]	27.3 ± 5.6 [19–43]	28.4 ± 6.1 [20–42]	24.5 ± 4.5 [20–38]	28.7 ± 6.9 [19–43]	30.2 ± 5.3 [20–39]	25.0 ± 3.3 [21–30]
**Xanthophyll Carotenoid Concentrations (Serum and Tissue)**						
Serum L, μmol/L	0.194 ± 0.096	0.184 ± 0.067	0.223 ± 0.116	0.188 ± 0.120	0.177 ± 0.108	0.187 ± 0.057
Serum Z, μmol/L	0.073 ± 0.029	0.074 ± 0.028	0.086 ± 0.038	0.071 ± 0.030	0.060 ± 0.022	0.073 ± 0.029
MPOV [range]	4553 ± 2095 [527–10,033]	5263 ± 1789 [2243–8861]	4793 ± 2885 [527–10,033]	3890 ± 1925 [1327–7649]	4277 ± 2115 [1027–8639]	4784 ± 1446 [2632–7880]
Skin Carotenoid Score	36,027 ± 12,458	37,970 ± 13,652	40,833 ± 15,063	34,656 ± 11,142	30,385 ± 12,065	36,538 ± 9173

^1^ Plus-minus values are means ± SD. There were no significant between-group differences at baseline except for BMI (*p* = 0.027). *P* values were based on chi-square and ANOVA or Kruskall–Wallis where appropriate. Abbreviations: L, lutein; Z, zeaxanthin; BMI, body mass index; y, years; MPOV, macular pigment optical volume. Interventions are as follows: Group 1, L (10 mg) + MZ (10 mg) + Z (2 mg) provided in one capsule; Group 2, L (10 mg) + MZ (10 mg) + Z (2 mg) provided in two capsules; Group 3, L (10 mg) + MZ (10 mg) + Z (2 mg) provided in DHA (430 mg) and EPA (90 mg) in two capsules; and Group 4, Ld (10 mg) + MZd (10 mg) + Zd (2 mg) provided in one capsule; or Group 5, placebo (sunflower oil).

**Table 2:** Carotenoid concentrations analysed per capsule interventions ^1^.

Carotenoid	Group 1	Group 2	Group 3	Group 4	Group 5
Lutein	9.42 ± 0.11	5.80 ± 0.19	4.48 ± 0.07	10.24 ± 0.54	0
*Meso*-zeaxanthin	13.06 ± 0.15	8.12 ± 0.27	6.49 ± 0.12	10.62 ± 0.61	0
Zeaxanthin	2.12 ± 0.03	1.38 ± 0.04	1.75 ± 0.03	1.98 ± 0.11	0
Dosage (capsule/day)	1	2	2	1	1
Total carotenoids ingested per day (mg)	24.60	30.60	25.44	22.84	0

^1^ Plus-minus values are means ± SD. Values are total carotenoid concentrations per capsule (mg). There were no significant between-group differences in change of L serum concentrations per gram taken (*p* = 0.419); change in Z, and MZ serum concentrations per gram taken were higher for Group 4 (*p* < 0.001). *P* values were based on chi-square and ANOVA or Kruskall-Wallis where appropriate. Bonferroni correction was performed for post-hoc analysis. Label claim for total nutrient concentrations were as follows: Group 1, L (10 mg) + MZ (10 mg) + Z (2 mg) provided in one capsule; Group 2, L (10 mg) + MZ (10 mg) + Z (2 mg) provided in two capsules; Group 3, L (10 mg) + MZ (10 mg) + Z (2 mg) provided in DHA (430 mg) and EPA (90 mg) in two capsules; and Group 4, Ld (10 mg) + MZd (10 mg) + Zd (2 mg) provided in one capsule; or Group 5, placebo (sunflower oil).

**Table 3:** Response in serum and tissue concentrations to different formulations of nutritional supplements with L, Z, and MZ compared to placebo ^1^.

Intervention	Outcome (μmol/L)					
		L	Z	MZ	MPOV	Skin
Group 1 (*n* = 11)	0 Mo	0.18 ± 0.07	0.07 ± 0.03	0	5263 ± 1789	37,970 ± 13,652
	6 Mo	0.61 ± 0.25	0.09 ± 0.03	0.06 ± 0.03	5943 ± 1567	52,303 ± 15,253
	Change	0.43 ± 0.22	0.02 ± 0.02	0.06 ± 0.03	680 ± 661	14,333 ± 8467
	*p* value ^2^	<0.001	0.007	<0.001	0.039	0.024
Group 2 (*n* = 14)	0 Mo	0.22 ± 0.12	0.09 ± 0.04	0	4793 ± 2885	40,833 ± 15,063
	6 Mo	0.56 ± 0.29	0.09 ± 0.02	0.04 ± 0.03	5802 ± 3254	48,571 ± 10,921
	Change	0.34 ± 0.29	0.00 ± 0.03	0.04 ± 0.03	1010 ± 914	7738 ± 9369
	*p* value ^2^	0.001	0.366	<0.001	0.006	0.543
Group 3 (*n* = 16)	0 Mo	0.19 ± 0.12	0.07 ± 0.03	0	3890 ± 1925	34,656 ± 11,142
	6 Mo	0.52 ± 0.29	0.09 ± 0.03	0.04 ± 0.03	4911 ± 1846	45,542 ± 10,750
	Change	0.33 ± 0.25	0.02 ± 0.03	0.04 ± 0.03	1021 ± 743	10,885 ± 7115
	*p* value ^2^	<0.001	0.008	<0.001	0.001	0.087
Group 4 (*n* = 13)	0 Mo	0.18 ± 0.11	0.06 ± 0.02	0	4277 ± 2115	30,385 ± 12,065
	6 Mo	0.58 ± 0.43	0.11 ± 0.05	0.16 ± 0.15	5331 ± 2061	47,718 ± 12,718
	Change	0.40 ± 0.38	0.05 ± 0.04	0.16 ± 0.15	1054 ± 680	17,333 ± 12,664
	*p* value ^2^	0.002	<0.001	0.001	0.001	0.012
Group 5 (*n* = 13)	0 Mo	0.19 ± 0.06	0.07 ± 0.03	0	4784 ± 1446	36,538 ± 9173
	6 Mo	0.18 ± 0.06	0.07 ± 0.02	0	4894 ± 1581	42,077 ± 9557
	Change	−0.01 ± 0.05	0.00 ± 0.01	0	110 ± 606	5539 ± 9125
	*p* value ^2^	-	-	-	-	-

^1^ Values are mean ± SD, L (indicates lutein serum concentrations, μmol/L); Z (indicates zeaxanthin serum concentrations, μmol/L); MZ (indicates *meso*-zeaxanthin serum concentrations, μmol/L); MPOV, macular pigment optical volume; Skin indicates skin carotenoid score. ^2^ Between-group differences comparing change from baseline in each intervention group with placebo were analysed with the use of an independent-sample *t*-test. Change was calculated as the difference from baseline. Interventions are as follows: Group 1, L (10 mg) + MZ (10 mg) + Z (2 mg) provided in one capsule; Group 2, L (10 mg) + MZ (10 mg) + Z (2 mg) provided in two capsules; Group 3, L (10 mg) + MZ (10 mg) + Z (2 mg) provided in DHA (430 mg) and EPA (90 mg) in two capsules; and Group 4, Ld (10 mg) + MZd (10 mg) + Zd (2 mg) provided in one capsule; or Group 5, placebo (sunflower oil).

**Table 4:** Effect of the diacetate formulation on serum and tissue response compared to the other formulations ^1^.

Outcome	Group 4 vs. Group 1		Group 4 vs. Group 2		Group 4 vs. Group 3	
	Difference in Change	*p*-Value	Difference in Change	*p*-Value	Difference in Change	*p*-Value
L	−0.027 (−0.297 to 0.244)	0.839	0.056 (−0.208 to 0.320)	0.666	0.070 (−0.175 to 0.315)	0.563
Z	0.033 (0.006 to 0.059)	0.018	0.045 (0.018 to 0.072)	0.002	0.030 (0.005 to 0.055)	0.019
MZ	0.109 (0.020 to 0.197)	0.019	0.124 (0.036 to 0.211)	0.009	0.126 (0.039 to 0.214)	0.008
MPOV	374 (−196 to 944)	0.187	45 (−598 to 687)	0.888	33 (−515 to 581)	0.903
SKIN	3000 (−6310 to 12,310)	0.511	9595 (811 to 18,379)	0.034	6448 (−1191 to 14,087)	0.095

^1^ Values are mean (95%CI). Abbreviations: L, lutein (indicates L serum concentrations, μmol/L); Z, zeaxanthin (indicates Z serum concentrations, μmol/L); MZ, *meso*-zeaxanthin (indicates MZ serum concentrations, μmol/L); MPOV, macular pigment optical volume; Skin indicates skin carotenoid score. The between-group differences were analysed with the use of an independent-sample t-test to compare group 4 against the other 3 active groups. Interventions are as follows: Group 1, L (10 mg) + MZ (10 mg) + Z (2 mg) provided in one capsule; Group 2, L (10 mg) + MZ (10 mg) + Z (2 mg) provided in two capsules; Group 3, L (10 mg) + MZ (10 mg) + Z (2 mg) provided in DHA (430 mg) and EPA (90 mg) in two capsules; and Group 4, Ld (10 mg) + MZd (10 mg) + Zd (2 mg) provided in one capsule; or Group 5, placebo (sunflower oil).

## Supplementary Materials

The following are available online at https://www.mdpi.com/2076-3921/9/8/767/s1, Figure S1: Representative chiral HPLC carotenoid profile of a capsule from intervention Group 2.
