# Supplementary material for: The Impact of Formulation on Lutein, Zeaxanthin, and meso-Zeaxanthin Bioavailability: A Randomised Double-Blind Placebo-Controlled Study
Source: Antioxidants (Basel). 2020 Aug 18;9(8):767. doi: 10.3390/antiox9080767 (PMC7463514; doi:10.3390/antiox9080767)
Supplement: Supplementary file 1 [file antioxidants-09-00767-s001.pdf]

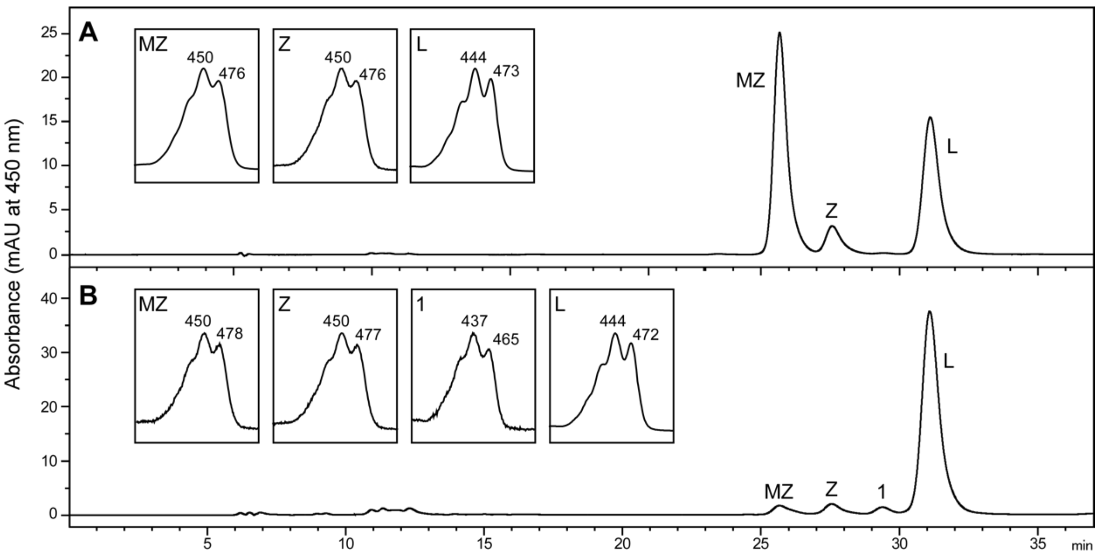

Figure S1. Representative chiral HPLC carotenoid profile of a capsule from intervention Group 2. (A) Analysis of the complete content of the capsule (8 mg of *meso*-zeaxanthin (MZ), 1 mg of zeaxanthin (Z) and 6 mg of lutein (L) microcrystals suspended in vegetable oil. (B) Analysis of the vegetable oil separated from the microcrystals (1, unidentified carotenoid).
